# The MAHB, the Culture Gap, and Some Really Inconvenient Truths

**DOI:** 10.1371/journal.pbio.1000330

**Published:** 2010-04-06

**Authors:** Paul R. Ehrlich

**Affiliations:** Stanford University, Stanford, California, United States of America

## Abstract

Humanity's failure to take adequate actions to stem a likely environmental collapse calls for extraordinary measures to understand and alter human behavior, argues Paul Ehrlich. His Millennium Assessment of Human Behavior (MAHB) aims to chart the path to a sustainable future.

**Figure pbio-1000330-g001:**
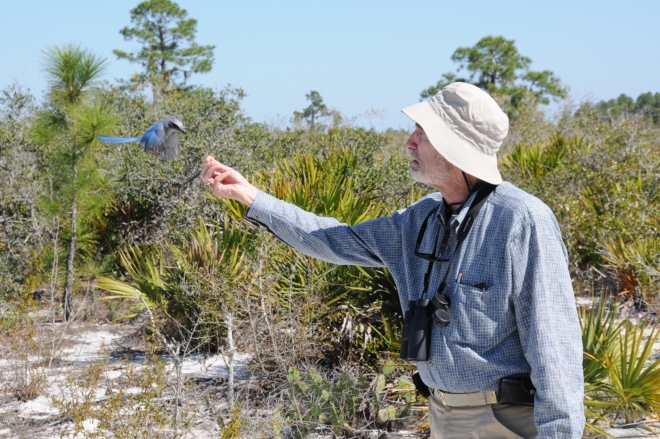
About the Author. **Paul Ehrlich is a professor of population studies at Stanford University (http://www.stanford.edu/group/CCB/Staff/Ehrlich.html). The Millennium Assessment of Human Behavior is his latest initiative to educate the public about the threats that uncontrolled human activities pose to the environment. A pioneer in the field of coevolution known for his long-term studies of the structure, dynamics, and genetics of natural butterfly populations, Ehrlich first sounded the alarm about the environmental impacts of overpopulation and resource exploitation in his 1968 book *The Population Bomb*. He is the recipient of numerous scientific awards, including the 2009 Ramon Margalef Award in Ecology and Environmental Sciences, which honors exceptional lifetime achievements or discoveries.**

[Fig pbio-1000330-g001]The human predicament—climate disruption, loss of biodiversity and ecosystem services, toxification of the planet, the potential impacts of nuclear war, and social and economic inequities that impede solutions to escalating environmental problems—has been amply described [1]. Although the steps needed to solve the predicament are clear, few have been taken—even as the situation steadily declines. The trend in greenhouse gas emissions has continued rapidly upward. The extermination of biodiversity and loss of natural services has proceeded unabated. The number of hungry people has hit an all-time high, which means that so has the number of immune-compromised individuals. That, combined with continued rapid population growth, increases the probabilities of vast epidemics [2]. In Asia, melting of the Himalayan water tower [3] and rising temperatures threaten the food supply of 1.6 billion people [4] whose countries are armed with nuclear weapons [5]. There also have been increasing signs of great toxic peril for humanity and its life-support systems, with a growing threat from the release of hormone-disrupting chemicals that could even be shifting the human sex ratio [6] and reducing sperm counts.

Despite the clear warnings about the predicament almost two decades ago from the scientific community [7],[8], precious little has been done. That's why a group of social and natural scientists and scholars in the humanities is starting the Millennium Assessment of Human Behavior (MAHB, pronounced “mob”). The admittedly ambitious aim is to change human behavior to avoid a collapse of global civilization.

The urgent need for this call to action is clear when you consider that efforts to address even the most publicized of environmental problems—climate disruption—have fallen far short. Fifteen international conferences have effected no significant change in the accumulation of greenhouse gases and no enforceable agreement yet to reverse the trend. How much failure is enough? Even if nations were to fulfill their recent pledges, catastrophic climate change might well be inevitable [9]. The climate challenge will persist over centuries or even millennia [10] and will require an urgent revision of humanity's energy mobilizing systems and of deforestation and other greenhouse gas (GHG)-releasing land uses. It will also necessitate a continual reworking of water-handling infrastructure to adjust to changing precipitation patterns that are vital to agriculture, as well as massive adjustments of human settlements as sea levels rise, among many other adaptations.

Whereas at least climate disruption is on the political agenda, most of the other issues are not, and public understanding of what drives environmental deterioration or, indeed, of natural phenomena in general is minimal. Few non-scientists are familiar with the basic idea that environmental damage is a product of population size, per capita consumption, and the sorts of technologies and social and economic systems that supply the consumption. A vast “culture gap” has developed over the past century or so between what our society knows and what each individual knows—a gap that has proven especially troubling when elected officials and other leaders have almost no knowledge of science [11].

That's one reason why the devastating environmental consequences of an ever-expanding human population have been largely ignored. Governments in many struggling poor countries fail to support family planning programs adequately, whereas those in the rich countries of Europe are irrationally encouraging higher fertility [12]. Few recognize that adding a billion people to the population in the future will cause more damage to humanity's critical life-support systems than did the most recent increment of a billion, as ever more scarce and remote resources must be tapped to support the newcomers.

Overconsumption by the rich is central to the deterioration of human life-support systems, but is ignored because most business economists, corporate executives, and politicians view it as an unalloyed good. To lead decent lives, at least two billion people are in dire need of *more* consumption, but extending American consumption patterns to even today's 6.8 billion people is not only unsustainable but likely a biophysical impossibility.

It would, sadly, take many decades for humane actions to produce significant changes in today's population trajectory. Yet, we know that consumption patterns can change virtually overnight, as demonstrated by the mobilizations and demobilizations connected with World War II. Enormous changes in production and consumption occurred in the United States in 4–5 years, and, during those years, Americans accepted rationing of gasoline, sugar, and meat. Given appropriate incentives, economies can be transformed extremely rapidly.

Undertaking a World War II–type mobilization, possibly lasting several times longer, to reduce GHG emissions fast and deal with the rest of the predicament would take vast political courage. The urgent need now is clearly not for more natural science (although in many areas it would be helpful) but rather for better understanding of human behaviors and how they can be altered to direct *Homo sapiens* onto a course toward a sustainable society, to muster that courage before it's too late. Indeed, the academic focus for solving the predicament needs to shift dramatically to the social sciences and the humanities. Understanding such things as how social norms are generated and how individual actions get translated into group behavior are, in my opinion, central to organizing a successful effort [13].

It is human *behavior*, toward one another and toward the planet that sustains us all, that requires rapid modification. The MAHB [14],[15] hopes to provide a basic mechanism to achieve this by (1) exposing society to the full range of “inconvenient truths” regarding population–environment–resource–ethics–power issues, (2) sponsoring a broad global discussion involving the greatest possible diversity of people, and (3) trying to close crucial parts of the culture gap.

We must humanely reduce the size of the global population, take steps to stop the growth of per-capita consumption among the rich (while increasing it among the poor), and face the need to gradually reduce the scale of the entire human physical economy. This will require developing mechanisms to force big corporations (including those in big agriculture and big pharma) to bear social responsibilities like the real individuals whose rights they legally want to assume [16]. Corporations are not an essential feature of capitalism, and, in any case, one of the most inconvenient truths is that if capitalism must depend on non-asymptotic perpetual growth of the physical economy, capitalism will disappear. Like it or not, the human enterprise simply must be constrained if it is to persist.

The MAHB intends to generate a global discussion of the human predicament, what people desire, and what goals are possible to achieve in a sustainable society. The MAHB also differs in seeking input from both the scholarly community and the general public on how to organize itself, and it will remain open to such input (see Box 1).

**Box 1. Negotiating the MAHB's path to change**

The Millennium Assessment of Human Behavior (MAHB, http://mahb.stanford.edu/.) aims to promote rapid change in human behavior to avoid the collapse of global civilization. The MAHB will be developed in association with scientists, scholars, and the general public. The MAHB, initiated at Stanford University, is still at a very preliminary stage. It now needs input to make the following decisions:

1. Who is the audience for the MAHB, and whom does it hope to influence?
2. How much should the MAHB critique current institutional and social practices and suggest directions for the necessary changes? Would success require new or highly altered institutions?
3. Has incrementalism by major institutions failed to deal with almost all the most serious environmental problems? Will it continue to fail?
4. Could conversations and publications nudge existing organizations to modify their behavior in a more sustainable direction, including helping others to become sustainable?
5. Should the MAHB be revolutionary and work with grassroots groups in an effort to compel governments and other organizations to take a more direct and effective course that would avoid a collapse of civilization?
6. How much should the MAHB focus on proposing routes to sustainability through large (often global) organizations and how much on encouraging experimentation at community and regional levels?
7. Should the MAHB give high priority to exploring potential scenarios for going forward to create a coherent plan for eliciting political, economic, and social behaviors to maintain human life-support systems and make civilization sustainable?
8. How can the critical parts of the culture gap be closed quickly?

I hope that as many readers of *PLoS Biology* as possible will get engaged in the MAHB, create discussion groups, and communicate with other discussion groups and the general public to jumpstart a global conversation and a mass movement. Those groups are already forming, one even at the middle-school level, and symposia and get-togethers focused on the MAHB are already scheduled for the annual meetings of the Ecological Society of America in Pittsburgh and the World Congress of Sociology in Sweden, both in the summer of 2010.

Within academia, I hope the MAHB can become the focus of badly needed new, coordinated efforts by social scientists and scholars in the humanities to help solve the human predicament. It will seek key points at which human behavior should be changed and evaluate the most humane ways to do it, finding new ones and working with old ones, ranging from Sabido soap operas (e.g., [17]) and proper “framing” of issues [18] to deliberative polling (e.g., [19]).

In relation to both outreach and research functions, the MAHB envisions establishing an “observatory” on behavior, gathering evidence from existing documents, established databases, and global stakeholders, and promoting new directions for outreach and new research projects. If funding can be found, the behavioral observatory would establish a MAHB-line (analogous to Medline), providing access to social science and humanities research relating to sustainability. It will have an interactive portal receiving and providing up-to-date information about particular environmental problems, human factors relating to these problems, and initiatives to deal with them.

The MAHB aims to organize a world megaconference in 2011 or 2012 that would initiate a continuing process, making the MAHB a semi-permanent, autonomous transnational institution. I emphasize “transnational,” as it should focus on relationships of people around the world with one another and their environments, and not “international,” which shifts the focus to between nation states, clearly obsolescent institutional structures. The MAHB is now at a preliminary stage; its nascent website has just been opened to the public. The need for input from people accustomed to working in the social sciences and humanities, in the media, in the business community, and so on, is obvious. If you are willing to get involved, go to http://mahb.stanford.edu/. There, you can join the effort to get humanity to do what is obviously required but usually deemed impractical. A global consensus on the most crucial behavioral issues is unlikely to emerge promptly from the MAHB or any other transnational effort. But, since the MAHB is envisioned as an ongoing flexible effort, not all the goals would need to be reached immediately. If the scientific diagnosis of humanity's approaching collision with the natural world is accurate (and I and my colleagues believe it is), what alternative is there to trying?
